# Optimal Choice as First-Line Therapy for Patients with Triple-Negative Breast Cancer: A Bayesian Network Meta-Analysis

**DOI:** 10.3390/curroncol29120718

**Published:** 2022-11-25

**Authors:** Yiqun Han, Jiayu Wang, Yun Wu, Hangcheng Xu, Yan Wang, Binghe Xu

**Affiliations:** Department of Medical Oncology, National Cancer Center/National Clinical Research Center for Cancer/Cancer Hospital, Chinese Academy of Medical Sciences and Peking Union Medical College, No. 17, Panjiayuan Nanli, Chaoyang District, Beijing 100021, China

**Keywords:** triple-negative breast cancer, metastases, first-line, therapy, network meta-analysis

## Abstract

To identify the advantageous therapy as the first-line treatment for patients with triple-negative breast cancer (TNBC). Randomized controlled trials were searched for on Medline, Embase, ClinicalTrials.gov, and the Cochrane Library between January 2001 and December 2021. The primary endpoint was progression-free survival (PFS) and secondary endpoints were overall survival (OS) and treatment-related adverse events (TRAEs). A Bayesian framework was applied to facilitate indirect comparisons, of which the outcomes were presented using cumulative ranking curve (SUCRA) values, synthesized hazard ratio, risk ratio, and 95% credible interval. A total of 3140 patients were identified. Pooled results of PFS revealed that chemotherapy plus AKT inhibitors (AKTi) was likely the most effective therapy among enrolled therapies (SUCRA = 91.6%), of which the result remained consistent in comparative analysis for OS. In addition, no significant difference was detected between PD-1/PD-L1 antibodies in patients, whereas the PD-1 inhibitors (PD-1i) regimen was advantageous over PD-L1 inhibitor (PD-L1i) therapy for PD-L1 positive TNBC. Concerning TRAEs, an apparent heterogeneity associated with safety profiles were denoted among enrolled agents. Chemotherapy plus AKTi was the most effective therapy with comparable safety profiles. Chemotherapy plus the anti-PD-1 regimen was advantageous over the combination therapy based on the PD-L1 blockade.

## 1. Introduction

Today, triple-negative breast cancer (TNBC) remains the most challenging molecular subtype of breast cancer, accounting for approximately between 15% and 20% of all new diagnoses of breast cancer, of which the inherent heterogeneity has not been fully elaborated [1]. With aggressive biologic behaviors, patients with TNBC tend to present an inferior clinical course and usually relapse at an early onset. Over the past decade, as the mainstay of treatment, chemotherapy, including paclitaxel or platinum, has been improving the prognosis of early TNBC patients. However, TNBC patients could rapidly cultivate resistance to cytotoxic agents and evolve to the advanced stage, which presents a poor prognosis, with a median overall survival (OS) of less than 20 months [2,3]; therefore, it is necessary to foster potential targets within this population.

With the in-depth understanding of cancer progression and drug resistance, novel agents toward intrinsic crosstalk or tumor immunity have been emerging and showing efficacious capability [4,5,6]. Chemotherapy has been challenged against the standard choice of the first-line treatment for metastatic TNBC. Considering there is no evidence from a directly comparative study, the optimal treatment for newly diagnosed metastatic TNBC could hardly be selected from current agents. Under this scenario, the indirectly comparative analysis provides a unique opportunity for protocol introduction and decision-making process. Herein, we carried out this network meta-analysis to identify the advantageous option as first-line therapy for patients with triple-negative breast cancer, aiming to assess the efficacy and safety profiles among current choices and curate evidence for clinical practice.

## 2. Materials and Methods

This study was conformed to Preferred Reporting Items for Systematic Reviews and Meta-analyses (PRISMA) and the PRISMA extension guidelines for network meta-analyses [7,8]. The protocol of this study has been prospectively registered with the International Platform of Registered Systematic Reviews and Meta-analysis Protocols (INPLASY) (Registration ID: INPLASY202180030).

### 2.1. Study Selection and Outcome Measures

We systematically searched for phase II/III randomized controlled trials (RCTs) published on Medline, Embase, ClinicalTrials.gov, and the Cochrane Library. Using searching strategies based on keywords such as ‘triple-negative breast cancer’, ‘metastases’, and ‘first-line’, studies published from January 2001 to June 2021 were successively evaluated. The screening strategy is listed in Figure S1. Studies reported on conference proceedings comprising the American Society of Clinical Oncology, European Society of Medical Oncology, and San Antonio Breast Cancer Symposium were also subjected to assessment for eligibility. We included studies with comparisons between chemotherapy and chemotherapy-based combination regimens and declared data on the primary outcomes. Studies were excluded if early TNBC patients were enrolled. The study selection was cut off on 31 December 2021.

The primary endpoint was progression-free survival (PFS), referring to the interval from randomization and first disease progression or death. Secondary endpoints were overall survival (OS), defined as the time between randomization and death by any cause. Defined by the 5th edition of Common Terminology Criteria for Adverse Events (CTCAE), treatment-related adverse events (TRAEs), consisting of rash, diarrhea, neuropathy, and neutropenia were also recorded to fully assess the safety profiles of therapies as the first-line treatment for TNBC patients. Programmed cell death ligand 1 (PD-L1) positive expression was referred to in accordance with reported definitions, which was the combined positive score of ≥ 1 for the PD-1 antibody and immune cell PD-L1 expression of ≥ 1% for the PD-L1 antibody.

### 2.2. Data Extraction and Quality Assessment

We extracted the first author, recruitment period, published year, ClinicalTrials.gov identifier, study phase, number of patients, treatment arms, PFS and OS parameters, estimates of hazard ratio (HR), and 95% confidence interval (CI) for PFS and OS, proportions of TRAEs. For one study, the final version for publication was included and the latest outcomes with the longest follow-up were extracted. For multiple subgroup analyses, we included the data on PFS and OS in the intention-to-treat population. Information was recorded by one investigator (HY) and concurrently reviewed by two investigators (XB and WJ). If any conflict existed, all the investigators would discuss it until discrepancies were resolved.

Quality assessment of individual studies, toward selection bias, performance bias, attribution bias, and detection bias, was carried out using the Cochrane Handbook for Systematic Reviews of Interventions (version 6.1, https://training.cochrane.org/handbook, accessed on 1 May 2021). Publication bias was assessed by the Begg and Egger tests in which *p* value < 0.10 was considered as significant bias. 

### 2.3. Statistical Analysis

Network meta-analysis was performed using R package gemtc (version 1.0-1) to assess the comparative efficacy among included treatment arms. The Bayesian framework was utilized to validate models with the Markov Chain Monte Carlo (MCMC) method using JAGS software (version 4.3.0). Synthetic estimates for survival outcomes were presented as HR and credible interval (CrI), while dichotomous data was displayed with risk ratio (RR) and 95% CI. Cochran Q Statistic and I^2^ Statistic were applied to measure statistical heterogeneity. Treatments were concurrently ranked based on posterior probabilities in addition to the surface under the cumulative ranking curve (SUCRA) values, ranging from 0% to 100%, which were in positive associations with the effective degree. All statistical analyses were conducted using Review Manager (version 5.4, https://training.cochrane.org/, accessed on 1 May 2021) R software (version 3.6.3, https://www.r-project.org/, accessed on 1 May 2021).

## 3. Results

**Figure 1:**
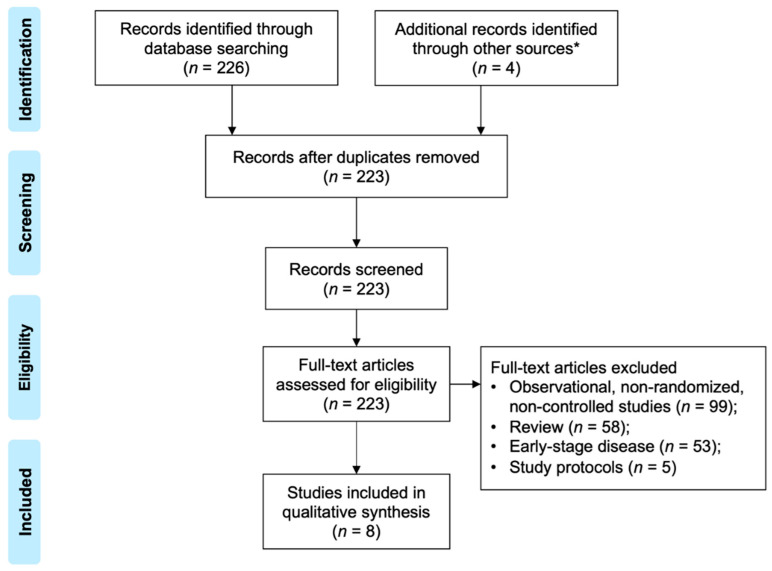
The diagram of study selection and identification. * Conference proceedings including the American Society of Clinical Oncology, European Society of Medical Oncology, and San Antonio Breast Cancer Symposium.

A total of 230 articles were screened and 8 studies were finally included [9,10,11,12,13,14,15,16,17], which involved 1868 and 1272 participants in the intervention and control group, respectively (Figure 1). The treatment adopted in the intervention groups comprised chemotherapy plus AKT inhibitors (*N* = 3, capivasertib, ipatasertib), anti-PD-1 antibodies (*N* = 1, pembrolizumab), anti-PD-L1 antibodies (*N* = 2, atezolizumab), and tyrosine kinase inhibitors (TKIs) (*N* = 2, cobimetinib, lapatinib), while the comparators were cytotoxin monotherapy. The characteristics of included trials are presented in Table S1. The respective bias of individual studies was acceptable (Figure S2), while the publication bias was not evaluated due to the insufficient number of the adopted literature [18]. 

### 3.1. Efficacy

**Figure 2:**
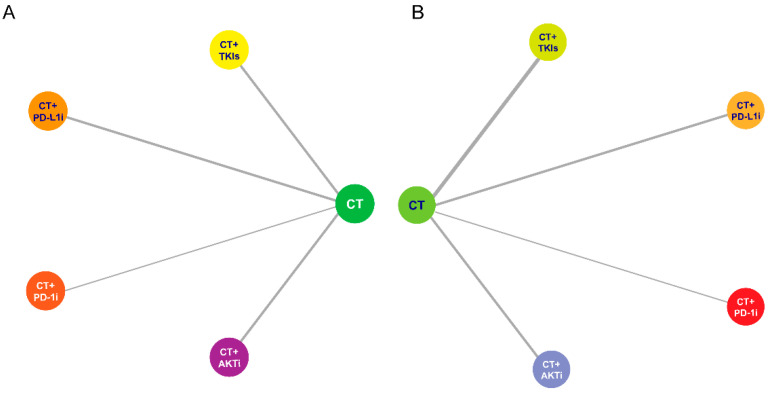
Network plot of the therapeutic options included in comparative analysis. (**A**) Therapeuutic options included for pooled PFS. (**B**) Therapeuutic options included for pooled OS. Abbreviation: AKTi, Protein kinase B inhibitor; CT, Chemotherapy; PD-1i, Programmed cell death protein 1 inhibitor; PD-L1i, Programmed cell death ligand 1 inhibitor; TKIs, Tyrosine kinase inhibitors.

**Figure 3:**
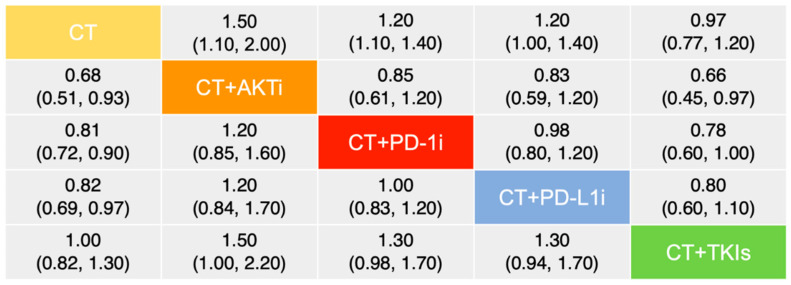
Comparative analysis of progression-free survival among selected therapies. Data are presented as hazard ratio and 95% credible interval. The upper right values display the top treatment compared to the bottom treatment. The lower left values demonstrate the converse comparisons. Abbreviation: AKTi, Protein kinase B inhibitor; CT, Chemotherapy; PD-1i, Programmed cell death protein 1 inhibitor; PD-L1i, Programmed cell death ligand 1 inhibitor; TKIs, Tyrosine kinase inhibitors.

Regarding PFS, there were 8 publications involving 3140 patients adopted into the comparative analysis (Figure 2A). Based on a fix-effect model (I^2^ = 1%), chemotherapy plus AKTi was estimated as the most effective therapeutic option (SUCRA = 91.6%), followed by chemotherapy plus anti-PD-1 (SUCRA = 67.2%) and anti-PD-L1 antibodies (SUCRA = 63.0%), and has a significant benefit over chemotherapy or plus TKIs (Figure 3; Table S2). In addition, no significant divergence could be detected in the benefits regarding PFS between anti-PD-1 and anti-PD-L1 antibodies. 

**Figure 4:**
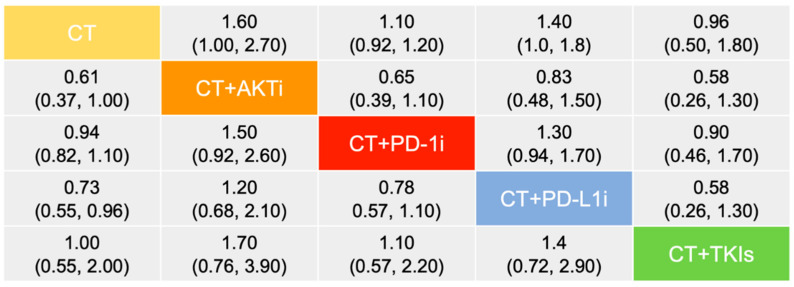
Comparative analysis of overall survival among adopted treatments. Data are presented as hazard ratio and 95% credible interval. The upper right values display the top treatment compared the bottom treatment. The lower left values demonstrate the converse comparisons. Abbreviation: AKTi, Protein kinase B inhibitor; CT, Chemotherapy; PD-1i, Programmed cell death protein 1 inhibitor; PD-L1i, Programmed cell death ligand 1 inhibitor; TKIs, Tyrosine kinase inhibitors.

With respect to OS, there were 5 studies containing 2630 participants enrolled in the indirect analysis (Figure 2B). Using a fix-effect model with acceptable heterogeneity (I^2^ = 7%), chemotherapy plus AKTi was likely the optimal treatment (SUCRA = 89.2%), of which the efficacy was of great superiority in comparison to other chemotherapy-based regimens, including anti-PD-L1 antibody, TKIs, or monotherapy; moreover, there were few benefits that could be observed among the later three therapies (Figure 4; Table S3). 

Additionally, comparative efficacy between PD-L1i and PD-1i was carried out for PD-L1 positive TNBC patients. Comparatively, the PD-li-containing regimen was advantageous over PD-L1 combined treatment regarding PFS (SUCRA, 87.6% vs. 62.3%) and OS (SUCRA, 94.6% vs. 50.2%), showing acceptable statistical heterogeneity within a fix-effect model (I^2^ = 1%) (Figures S3 and S4; Tables S4 and S5).

### 3.2. Safety

Treatment-related adverse events (TRAEs), consisting of rash, diarrhea, neutropenia, and neuropathy, were adopted to sensibly identify the most favorable therapy among included treatments. Results of comparative analysis suggested that chemotherapy plus TKIs could significantly aggravate the rate of rash compared to the other combination treatments (SUCRA = 9.3%), followed by AKTi-containing regimens (SUCRA = 32.6%), while the combination with anti-PD-L1 antibody showed that it might not add an apparent risk of rash for TNBC patients (Figure S5 and Table S6). 

Increasing risk of diarrhea could be observed in the TNBC patients when receiving chemotherapy in combination with AKTi (SUCRA = 12.3%) and TKIs (SUCRA = 27.3%), while a comparable incidence was suggested between chemotherapy and the combination with anti-PD-L1 antibody. Concerning diarrhea, it indicated that patients would suffer the foremost from receipt of chemotherapy plus anti-PD-L1 antibody (SUCRA = 23.2%) and AKTi (SUCRA = 39.4%), while the TKI might not exert additional risk of diarrhea (Figure S6; Table S7). This proportion of results was similar to those from neuropathy. Namely, chemotherapy in combination with AKTi could exacerbate the rate of neuropathy to the most extent (SUCRA = 20.9%), followed by anti-PD-L1 antibody (SUCRA = 36.5%) (Figure S7; Table S8). For neutropenia, a dramatically higher incidence could be recorded in patients administrated with chemotherapy plus PD-L1i (SUCRA = 23.2%) and AKTi (SUCRA = 39.4%), while the TKI-containing regimen provided the least risk of neutropenia in TNBC patients (Figure S8; Table S9). 

## 4. Discussion

Although novel agents have been arising and have demonstrated clinical efficacy over recent years, discrepancies do exist among the current options with no direct comparisons. Moreover, given the inferior prognosis of metastatic TNBC patients, the evaluation for the optimal therapy for this challenging subpopulation is urgent. In this study, we firstly assessed the efficacy and safety profiles of current therapeutic options as the first-line treatment for advanced TNBC. Through our analyses, it was demonstrated that chemotherapy plus AKTi was as the optimal treatment, and chemotherapy plus PD-1i tended to be more effective than PD-L1i combination treatment in PD-L1 positive patients. In addition, chemotherapy combination therapies have universally increased the risk of toxicity, while TRAEs were specifically associated with different treatment options. Taking into account these findings, more balance will be exerted between ‘for’ and ‘against’ when assessing the superior first-line therapy for TNBC patients in clinical practice. 

The survival parameter was always the leading consideration in adopting an intervention. In the presented study, we primarily focused on the PFS and analyzed the comparative effectiveness among enrolled treatments. Pooled analysis demonstrated that chemotherapy combination therapies were consistently more effective than chemotherapy alone. Subsequently, treatment arms successively underwent indirect comparative analyses through Bayesian algorithms, of which the results suggested that AKTi plus chemotherapy was likely the most favorable therapy as the first-line treatment for TNBC patients. This proportion of results were consistent and revealed that AKTi-based treatment could improve the OS for advanced TNBC patients to the utmost extent. Although the reliability could be inevitably hampered due to the immature data enrolled, the OS gains associated with AKTi-based treatment were strong evidence for superior treatment assessment in our study. Certainly, the overall prognosis tended to reflect on collective effects, including post-antineoplastic therapies, dynamic molecular alterations, and individual response, and whether improvement in PFS could be translated into OS gains remained controversial [19,20]. Moreover, considering the genomic profiles of participants, this therapeutic option might be more effective in TNBC with alternations in the PIK3CA/AKT1/PTEN pathway. Increasing evidence should be curated from individual data for lasting benefits in long-term survival. 

Notably, our research originally focused on the comparative efficacy between PD-1i and PD-L1i combination therapies for PD-L1 positive TNBC patients. It was revealed that the PD-1i-containing regimen likely provided more survival benefits than PD-L1-based treatment, of which the effectiveness of both was significantly greater than chemotherapy alone. Indeed, there were some researchers that paid attention to the difference among immune checkpoint inhibitors and reported that anti-PD-1 therapy was of a more effective profile [21,22]. However, few current studies have focused on the breast cancer population. From this perspective, our research firstly showed that immune checkpoint inhibitors could constantly improve the survival outcome in the entire breast cancer population. More importantly, we revealed that the efficacy was comparable between PD-1/PD-L1i in the entire population, while PD-1i-based therapy was likely the better choice as first-line treatment in PD-L1 positive breast cancer. Obviously, this was the first evidence for practitioners to select the immune checkpoint blockade for breast cancer patients with PD-L1 positive expression. 

The safety profile was another indispensable indicator for the introduction of therapeutic protocols and, therefore, we also took full account the comparative risk of the leading TRAEs reported in the included studies. Resembling the high incidence in TKIs-based treatment reported by previous studies [23,24], increased risk of rash was associated with TKIs plus chemotherapy. As a major concern associated with cytotoxins, neutropenia constitutes the most frequent reason for dose reduction or even therapy discontinuation [25]. Among the selected agents, it seemed that chemotherapy plus PD-L1i, followed by chemotherapy plus AKTi, added the foremost risk to TNBC patients. Considering the cytotoxins adopted in the majority of clinical trials were taxanes, of which neuropathic toxicity was a remarkable drug-induced side effect [26], the incidence of peripheral neuropathy was also successively assessed. Results of comparisons suggested that the AKTi-containing regimen likely presents the foremost risk of peripheral neuropathy, which was consistent with the result for diarrhea. Despite a slightly higher tendency, especially in peripheral neuropathy and diarrhea, which could be detected in the AKTi-based therapy, no tremendous significance was demonstrated. We supposed that it was comparable overall and required further validation. On top of that, profound heterogeneity should be noticed when evaluating the safety profiles of the treatment options. For instance, the AKTi was reported to aggravate the incidence of hyperglycemia [27,28], which was seldom observed among other therapeutic choices. With the rapid pace of advances in cancer immunotherapy, the immune-related adverse event was a notable effect following the immune checkpoint block, which we cannot ignore [29]. We concluded that specific factors, such as drug adverse profiles as well as baseline underlying disease, were supposed to influence individual-based protocols.

Results of this study should be deliberated due to the following limitations. For starters, this study was entirely based on the publications instead of individual data, which could result in inevitable bias. For example, the heterogenous survival outcomes have been posted by different clinical trials regarding AKTi in TNBC, requiring further assessment for confounding factors and prospective outcomes. Next, as the comparator, cytotoxic agents were mainly paclitaxel or nab-paclitaxel, of which the efficacy could be varied, and the clinical efficacy of other cytotoxins, such as platinum or capecitabine, cannot be fully elaborated in this study, potentially weakening the power of the findings [30,31,32]. Moreover, it was acknowledged that approximately 90% of TNBC would acquire inherent alterations following chemotherapy resistance and constituted targets for current treatments [33], such as PI3K inhibitors [12,13], PARP inhibitors [34], and MEK inhibitors [9]. However, we could not take into consideration the molecular profiles of eligible patients given the heterogeneity and scarcity among the publications. Lastly, the definition criteria of PD-L1 positive was not identical for PD-1i or PD-L1i, of which the results were applied with discretion. Considering our study was the first study to evaluate the paradigm of first-line treatment for TNBC, these findings could pave the way for the following studies for solid reference to prospective clinical practice.

## 5. Conclusions

In sum, this was the first study to evaluate the superior first-line treatment, toward both efficacy and safety profiles, for TNBC patients. Chemotherapy plus AKTi was likely the optimal therapeutic option, yet the toxicities should be considered based on individual heterogeneity. Patients with PD-L1 positive advanced TNBC could increasingly benefit from chemotherapy plus anti-PD-1 rather than anti-PD-L1 therapy. Population studies based on individual data are warranted in the future.

## Data Availability

Data supporting reported results are all included in the main text and supplementary materials.

## Supplementary Materials

The following supporting information can be downloaded at: https://www.mdpi.com/article/10.3390/curroncol29120718/s1, Table S1. Characteristics of enrolled studies for network meta-analysis; Table S2. Ranking probability of included treatments regarding progression-free survival; Table S3. Ranking probability of included treatments regarding overall survival; Table S4. Ranking probability of progress-free survival in PD-L1 positive patients; Table S5. Ranking probability of overall survival in PD-L1 positive patients; Table S6. Ranking probability of included treatments regarding rash; Table S7. Ranking probability of included treatments regarding diarrhea; Table S8. Ranking probability of included treatments regarding peripheral neuropathy; Table S9. Ranking probability of included treatments regarding neutropenia; Figure S1. Search strategy for literature selection; Figure S2. Quality assessment of individual study; Figure S3. Comparative analysis for progression-free survival in PD-L1 positive patients; Figure S4. Comparative analysis for overall survival in PD-L1 positive patients; Figure S5. Comparative analysis for the risk of rash among identified treatments; Figure S6. Comparative analysis for the risk of diarrhea among identified treatments. Figure S7. Comparative analysis for the risk of neuropathy among identified treatments. Figure S8. Comparative analysis for the risk of neutropenia among identified treatments.
