# Supplementary material for: Optimal Choice as First-Line Therapy for Patients with Triple-Negative Breast Cancer: A Bayesian Network Meta-Analysis
Source: Curr Oncol. 2022 Nov 25;29(12):9172–80. doi: 10.3390/curroncol29120718 (PMC9777258; doi:10.3390/curroncol29120718)
Supplement: Supplementary file 1 [file curroncol-29-00718-s001.zip › curroncol-2002170-supplementary.pdf]

Current Oncology

MDPI

Supplementals

Optimal Choice as First-Line Therapy for Patients with Triple-Negative Breast Cancer: A Bayesian Network Meta-Analysis

Yiqun Han, Jiayu Wang *, Yun Wu, Hangcheng Xu, Yan Wang and Binghe Xu *

Table S1. Characteristics of enrolled studies for network meta-analysis.

Author.	Recruitment period	Published year	ClinicalTrials.gov Identifier	Phase	No. of Patients		Treatment		Median PFS		Median OS	
					Intervention	Control	Intervention	Control	mo.	Hazard ratio	mo.	Hazard ratio
Adam Brufsky et al. [1]	2015.03-2016.10	2021	NCT02322814	II	47	43	Cobimetinib-paclitaxel	Paclitaxel	5.5 vs. 3.8	0.73 (95% CI 0.43-1.24)	16.0 vs. 19.6	1.05 (95% CI 0.55-2.01)
David Miles et al. [2]	2017.08-2019.09	2021	NCT03125902	III	431	220	Atezolizumab-paclitaxel	Paclitaxel	5.7 vs. 5.6	0.82 (95% CI 0.68-0.98)	22.8 vs. 19.2	1.12 (95% CI 0.88-1.43)
Javier Cortes et al. [3]	2017.01-2018.06	2020	NCT02819518	III	566	281	Pembrolizumab-CT	CT	7.5 vs. 5.6	0.82 (95% CI 0.69-0.97)	23.0 vs. 16.1	0.73 (95% CI 0.55-0.95)
Peter Schmid et al. [4]	2014.05-2017.06	2019	NCT03997123	II	70	70	Capivasertib-paclitaxel	Paclitaxel	5.9 vs. 4.2	0.74 (95% CI 0.50-1.08)	19.1 vs. 12.6	0.61 (95% CI 0.37-0.99)
Sung-Bae Kim et al. [5]	2014.09-2016.02	2017	NCT02162719	II	62	62	Ipatasertib-paclitaxel	Paclitaxel	6.2 vs. 4.9	0.60 (95% CI 0.37-0.98)	/	/
Rebecca Dent et al. [6,7]	2018.02-2020.04	2021	NCT03337724	III	170	85	Ipatasertib-paclitaxel	Paclitaxel	7.4 vs. 6.1	1.02 (95% CI 0.71-1.45)	/	/
Richard Finn et al. [8]	2004.01-2006.10	2009	NCT00075270	III	71	60	Lapatinib-paclitaxel	Paclitaxel	4.6 vs. 4.8	1.25 (95% CI 0.85-1.83)	/	/
Peter Schmid et al. [9]	2015.06-2017.05	2019	NCT02425891	III	451	451	Atezolizumab-nab-paclitaxel	nab-paclitaxel	7.2 vs. 5.5	0.80 (95% CI 0.69-0.92)	21.0 vs. 18.7	0.86 (95% CI 0.72-1.02)

Table S2. Ranking probability of included treatments regarding progression-free survival.

Treatment	SUCRA	Rank1	Rank2	Rank3	Rank4	Rank5
CT+AKTi	91.6%	78.67%	11.11%	8.45%	1.39%	0.39%
CT+PD-L1i	67.2%	10.24%	50.24%	37.47%	2.06%	0.01%
CT+PD-1i	63.0%	10.74%	36.84%	46.56%	5.29%	0.59%
CT	15.4%	0.00%	0.00%	1.14%	59.38%	39.49%
CT+TKIs	12.9%	0.37%	1.83%	6.39%	31.89%	59.54%

Table S3. Ranking probability of included treatments regarding overall survival.

Treatment	SUCRA	Rank1	Rank2	Rank3	Rank4	Rank5
CT+AKTi	89.2%	70.53%	20.85%	5.51%	2.00%	1.12%
CT+PD-1i	76.0%	23.25%	60.67%	13.80%	1.73%	0.56%
CT+PD-L1i	38.3%	0.37%	6.31%	49.01%	35.00%	9.32%
CT+TKIs	26.7%	5.85%	11.76%	18.76%	8.20%	55.44%
CT	19.8%	0.01%	0.42%	12.93%	53.08%	33.57%

Table S4. Ranking probability of progress-free survival in PD-L1 positive patients.

Treatment	SUCRA	Rank1	Rank2	Rank3
PD-1i+CT	87.6%	75.31%	24.56%	0.14%
PD-L1i+CT	62.3%	24.70%	75.24%	0.07%
CT	0.1%	0.00%	0.21%	99.80%

Table S5. Ranking probability of overall survival in PD-L1 positive patients.

Treatment	SUCRA	Rank1	Rank2	Rank3
PD-1i+CT	94.6%	90.31%	8.51%	1.19%
PD-L1i+CT	50.2%	9.55%	81.20%	9.25%
CT	5.3%	0.15%	10.30%	89.56%

Table S6. Ranking probability of included treatments regarding rash.

Treatment	SUCRA	Rank1	Rank2	Rank3	Rank4
CT+TKIs	9.3%	1.91%	2.66%	16.84%	78.60%
CT+AKTi	32.6%	4.41%	7.96%	68.59%	19.04%
CT+PD-L1i	75.0%	39.89%	47.34%	10.67%	2.11%
CT	83.1%	53.79%	42.04%	3.91%	0.26%

Table S7. Ranking probability of included treatments regarding diarrhea.

Treatment	SUCRA	Rank1	Rank2	Rank3	Rank4
CT+AKTi	12.3%	0.81%	2.17%	30.11%	66.92%
CT+TKIs	27.3%	3.80%	5.56%	59.28%	31.37%
CT+PD-L1i	75.2%	36.69%	53.93%	7.78%	1.61%
CT	85.2%	58.71%	38.35%	2.84%	0.11%

Table S8. Ranking probability of included treatments regarding peripheral neuropathy.

Treatment	SUCRA	Rank1	Rank2	Rank3	Rank4
CT+AKTi	20.9%	4.99%	12.12%	23.64%	59.27%
CT+PD-L1i	36.5%	8.07%	21.55%	42.35%	28.04%
CT	61.9%	16.32%	56.08%	24.46%	3.15%
CT+TKIs	80.7%	70.63%	10.26%	9.57%	9.55%

Table S9. Ranking probability of included treatments regarding neutropenia.

Treatment	SUCRA	Rank1	Rank2	Rank3	Rank4
CT+PD-L1i	23.2%	5.08%	13.81%	26.90%	54.22%
CT+AKTi	39.4%	11.48%	28.79%	26.28%	33.46%
CT	51.0%	6.53%	46.72%	40.02%	6.74%
CT+TKIs	86.3%	76.92%	10.70%	6.81%	5.58%

01. triple-negative breast cancer/
02. triple-negative breast cancer.tw.
03. 1 or 2
04. first-line
05. metastasis/
06. metastasis.tw.
07. 5 or 6
08. 3 and 4 and 7
09. randomized controlled trials/
10. randomized-controlled-trial.pt
11. controlled-clinical-trial.pt
12. random allocation/
13. 9 or 10 or 11 or 12
14. comparative study/
15. exp evaluation studies/
16. follow-up studies/
17. prospective studies/
18. 14 or 15 or 16 or 17
19. 8 and 13 and 18

Figure S1. Search strategy for literature selection.

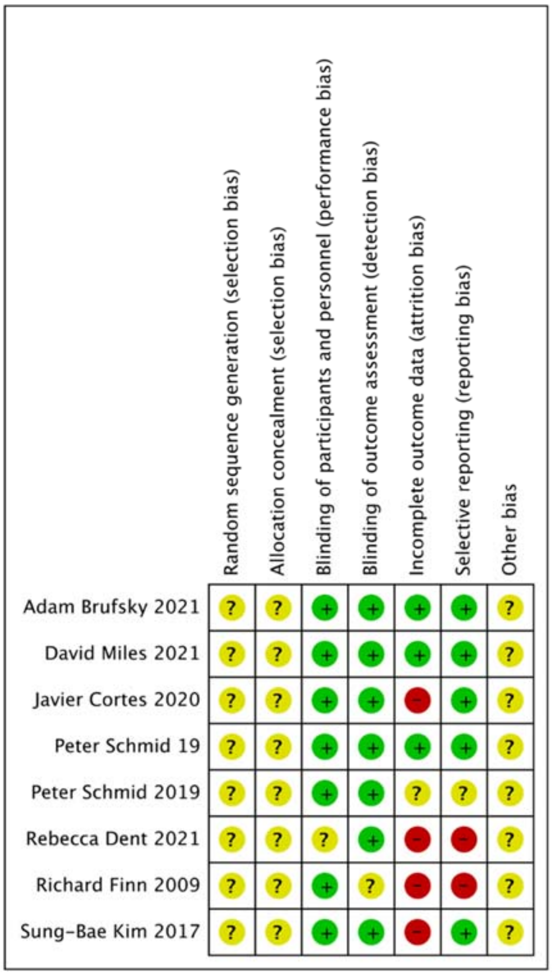

Figure S2. Quality assessment of individual study. Green represents low risk of bias, yellow represents unclear risk of bias, and red represents high risk of bias.

**PD-L1i+CT**	1.10 (0.86, 1.40)	0.80 (0.71, 0.92)
0.92 (0.73, 1.20)	**PD-1i+CT**	0.74 (0.61, 0.90)
1.20 (1.10, 1.40)	1.40 (1.10, 1.60)	**CT**

Figure S3. Comparative analysis for progression-free survival in PD-L1 positive patients.

**PD-L1i+CT**	0.81 (0.59, 1.10)	0.73 (0.56, 0.96)
1.2 (0.90, 1.70)	**PD-1i+CT**	0.90 (0.77, 1.10)
1.4 (1.0, 1.80)	1.1 (0.95, 1.30)	**CT**

Figure S4. Comparative analysis for overall survival in PD-L1 positive patients.

**CT**	0.56 (0.24, 1.30)	0.95 (0.44, 2.00)	0.32 (0.08, 1.00)
1.80 (0.77, 4.20)	**CT+AKTi**	1.70 (0.52, 5.30)	0.33 (0.07, 1.40)
1.00 (0.49, 2.30)	0.58 (0.19, 1.90)	**CT+PD-L1i**	0.57 (0.11, 2.50)
3.10 (0.96, 13.0)	1.80 (0.41, 9.30)	3.00 (0.73, 14.00)	**CT+TKI**

Figure S5. Comparative analysis for the risk of rash among identified treatments.

**CT**	0.30 (0.11, 0.76)	0.93 (0.36, 2.30)	0.39 (0.09, 1.40)
3.30 (1.30, 9.10)	**CT+AKTi**	3.10 (0.82, 12.00)	1.30 (0.24, 6.70)
1.10 (0.43, 2.80)	0.32 (0.08, 1.20)	**CT+PD-L1i**	0.42 (0.08, 2.10)
2.60 (0.70, 11.00)	0.77 (0.15, 4.20)	2.40 (0.49, 13.00)	**CT+TKI**

Figure S6. Comparative analysis for the risk of diarrhea among identified treatments.

**CT**	0.74 (0.41, 1.30)	0.86 (0.55, 1.40)	1.40 (0.52, 3.70)
1.30 (0.76, 2.40)	**CT+AKTi**	1.20 (0.57, 2.50)	1.90 (0.59, 5.80)
1.20 (0.71, 1.80)	0.87 (0.41, 1.80)	**CT+PD-L1i**	1.60 (0.54, 4.80)
0.72 (0.27, 1.90)	0.53 (0.17, 1.70)	0.63 (0.21, 1.80)	**CT+TKI**

Figure S7. Comparative analysis for the risk of neuropathy among identified treatments.

**CT**	0.90 (0.30, 2.40)	0.77 (0.32, 1.80)	1.90 (0.51, 7.70)
1.10 (0.42, 3.40)	**CT+AKTi**	0.84 (0.24, 3.50)	2.10 (0.41, 12.00)
1.30 (0.56, 3.10)	1.20 (0.28, 4.20)	**CT+PD-L1i**	2.50 (0.49, 13.00)
0.52 (0.13, 2.00)	0.47 (0.08, 2.40)	0.40 (0.08, 2.00)	**CT+TKI**

Figure S8. Comparative analysis for the risk of neutropenia among identified treatments.

References

1. Brufsky, A.; Kim, S.; Zvirbule, .; Eniu, A.; Mebis, J.; Sohn, J.; Wongchenko, M.; Chohan, S.; Amin, R.; Yan, Y.; et al. A phase II randomized trial of cobimetinib plus chemotherapy, with or without atezolizumab, as first-line treatment for patients with locally advanced or metastatic triple-negative breast cancer (COLET): primary analysis. *Ann. Oncol.* **2021**, *32*, 652–660, https://doi.org/10.1016/j.annonc.2021.01.065.
2. Miles, D.; Gligorov, J.; André, F.; Cameron, D.; Schneeweiss, A.; Barrios, C.; Xu, B.; Wardley, A.; Kaen, D.; Andrade, L.; et al. Primary results from IMpassion131, a double-blind, placebo-controlled, randomised phase III trial of first-line paclitaxel with or without atezolizumab for unresectable locally advanced/metastatic triple-negative breast cancer. *Ann. Oncol.* **2021**, *32*, 994–1004, https://doi.org/10.1016/j.annonc.2021.05.801.
3. Cortes, J.; Cescon, D.W.; Rugo, H.S.; Nowecki, Z.; Im, S.-A.; Yusof, M.M.; Gallardo, C.; Lipatov, O.; Barrios, C.H.; Holgado, E.; et al. Pembrolizumab plus chemotherapy versus placebo plus chemotherapy for previously untreated locally recurrent inoperable or metastatic triple-negative breast cancer (KEYNOTE-355): a randomised, placebo-controlled, double-blind, phase 3 clinical trial. *Lancet* **2020**, *396*, 1817–1828, https://doi.org/10.1016/s0140-6736(20)32531-9.
4. Schmid, P.; Abraham, J.; Chan, S.; Wheatley, D.; Brunt, A.M.; Nemsadze, G.; Baird, R.D.; Park, Y.H.; Hall, P.S.; Perren, T.; et al. Capivasertib Plus Paclitaxel Versus Placebo Plus Paclitaxel As First-Line Therapy for Metastatic Triple-Negative Breast Cancer: The PAKT Trial. *J. Clin. Oncol.* **2020**, *38*, 423–433, doi:10.1200/jco.19.00368.
5. Kim, S.-B.; Dent, R.; Im, S.-A.; Espié, M.; Blau, S.; Tan, A.R.; Isakoff, S.J.; Oliveira, M.; Saura, C.; Wongchenko, M.J.; et al. Ipatasertib plus paclitaxel versus placebo plus paclitaxel as first-line therapy for metastatic triple-negative breast cancer (LOTUS): a multi-centre, randomised, double-blind, placebo-controlled, phase 2 trial. *Lancet Oncol.* **2017**, *18*, 1360–1372, doi:10.1016/s1470-2045(17)30450-3.

6. Dent R, Kim S-B, Oliveira M, Barrios C, O'Shaughnessy J, Isakoff SJ, et al. Abstract GS3-04: Double-blind placebo (PBO)-controlled randomized phase III trial evaluating first-line ipatasertib (IPAT) combined with paclitaxel (PAC) for PIK3CA/AKT1/PTEN-altered locally advanced unresectable or metastatic triple-negative breast cancer (aTNBC): primary results from IPATunity130 Cohort A. Cancer Research. 2021;81:GS3-04-GS3-.
7. Dent, R.; Oliveira, M.; Isakoff, S.J.; Im, S.-A.; Espié, M.; Blau, S.; Tan, A.R.; Saura, C.; Wongchenko, M.J.; Xu, N.; et al. Final results of the double-blind placebo-controlled randomized phase 2 LOTUS trial of first-line ipatasertib plus paclitaxel for inoperable locally advanced/metastatic triple-negative breast cancer. *Breast Cancer Res. Treat.* **2021**, *189*, 377–386, https://doi.org/10.1007/s10549-021-06143-5.
8. Finn, R.S.; Press, M.F.; Dering, J.; Arbushites, M.; Koehler, M.; Oliva, C.; Williams, L.S.; Di Leo, A. Estrogen Receptor, Progesterone Receptor, Human Epidermal Growth Factor Receptor 2 (HER2), and Epidermal Growth Factor Receptor Expression and Benefit From Lapatinib in a Randomized Trial of Paclitaxel With Lapatinib or Placebo As First-Line Treatment in HER2-Negative or Unknown Metastatic Breast Cancer. *J. Clin. Oncol.* **2009**, *27*, 3908–3915, https://doi.org/10.1200/jco.2008.18.1925.
9. Schmid, P.; Rugo, H.S.; Adams, S.; Schneeweiss, A.; Barrios, C.H.; Iwata, H.; Diéras, V.; Henschel, V.; Molinero, L.; Chui, S.Y.; et al. Atezolizumab plus nab-paclitaxel as first-line treatment for unresectable, locally advanced or metastatic triple-negative breast cancer (IMpassion130): updated efficacy results from a randomised, double-blind, placebo-controlled, phase 3 trial. *Lancet Oncol.* **2019**, *21*, 44–59, https://doi.org/10.1016/s1470-2045(19)30689-8.
